# Theophylline exhibits anti-cancer activity *via* suppressing SRSF3 in cervical and breast cancer cell lines

**DOI:** 10.18632/oncotarget.21464

**Published:** 2017-10-03

**Authors:** Yung-Lung Chang, Yu-Juei Hsu, Ying Chen, Yi-Wen Wang, Shih-Ming Huang

**Affiliations:** ^1^ Department of Biochemistry, National Defense Medical Center, Taipei, Taiwan, Republic of China; ^2^ Division of Nephrology, Department of Medicine, Tri-Service General Hospital, National Defense Medical Center, Taipei, Taiwan, Republic of China; ^3^ Department of Biology and Anatomy, National Defense Medical Center, Taipei, Taiwan, Republic of China

**Keywords:** methylxanthine, caffeine, theophylline, SRSF3, p53

## Abstract

Caffeine, theophylline, and theobromine are the most well-known members of methylxanthines. Caffeine-induced serine/arginine-rich splicing factor 2, SRSF2, and SRSF3 are required for the alternative splicing of a subset of cancer-associated genes. However, it remains to be investigated whether and how theophylline and theobromine as well as caffeine exert their antitumor effects through mediating the alternative splicing process. Here, we reveal that theophylline down-regulated *SRSF3* expression and switched *p53* from alpha into a beta isoform as caffeine did in HeLa and MCF-7 cells via the reverse-transcriptase polymerase chain reaction and Western blot analysis. Further functional studies show that theophylline induced cellular apoptosis, senescence, and decreased colony formation. Interestingly, theophylline had a suppressive effect on cellular proliferation, whereas caffeine enhanced cellular proliferation rates via the 5-bromo-2-deoxyuridine analysis. Theophylline and caffeine had no effect on MCF-10A cells, which is a normal breast cell line. Our results provide an insight that theophylline as well as caffeine could be repurposed as antitumor leading compounds via the downregulation of splicing factor SRSF3 and its target genes.

## INTRODUCTION

Human pre-mRNA transcripts are alternatively spliced by specific trans-factors, including serine/arginine-rich splicing factors (SRSFs), but the regulatory mechanism of SRSFs for different biological processes is not well studied [1–5]. Recent studies have demonstrated that caffeine-induced *SRSF2* and *SRSF3* are required for the alternative splicing of the tumor suppressor genes, including *Krüppel-like factor 6*, *KLF6,* and *p53*, respectively [6, 7]. Caffeine, a 1,3,7-trimethylxanthine derivative, has been reported to induce alternative splicing for a subset of cancer-associated genes, such as *p53*, *pyruvate kinase M1*/*M2*, *hypoxia-inducible factor-1α/2α*, (*HIF-1α*/*2α*), and other pre-mRNAs [7–10]. Caffeine, theophylline (1,3-dimethylxanthine) and theobromine (3,7-dimethylxanthine) are the most well-known members of methylxanthines [11]. However, it remains to be investigated as to the functional role of natural methylxanthines during the alternative splicing process for antitumor functions [4].

Adenosine receptor modulation, phosphodiesterase inhibition, calcium level regulation, and modulation of gamma-aminobutyric acid receptors are the four main mechanisms proposed to mediate the pharmacological activity of methylxanthine at the cellular level [11]. Theophylline and theobromine, as well as caffeine, are recently reported to hold antitumor potential [12–15]. Theophylline has been used to treat airway diseases for more than 80 years. It was originally used as a bronchodilator, and more recently it has been shown to have anti-inflammatory effects, depending on the concentration [16]. Selective molecular mechanisms of action have been proposed for theophylline including nuclear factor-κB (NF-κB) inhibition, histone deacetylase 2 activity augmentation, and interleukine-10 secretion [11, 17]. A recent study showed that theophylline should be considered a potential anti-cancer drug in combination with other chemotherapeutic drugs in lung cancer patients [15].

Theobromine, a metabolite of caffeine, is less active than caffeine via the pharmacological assays, including the affinity for the adenosine receptor and less efficacious as a phosphodiesterase inhibitor [11]. More recently, several potential beneficial actions of theobromine have been described, including a capacity to enhance high-density lipoprotein cholesterol concentrations in healthy volunteers or increase NAD^+^/SirT1 activity for renoprotection under diabetic conditions [18, 19]. Theobromine, as well as caffeine, is now known to cross the blood brain barrier [20] and it inhibits glioblastoma cell proliferation via the negative regulation of phosphodiesterase 4, extracellular signal-regulated kinases, protein kinase B/mechanistic target of rapamycin, and NF-κB [15].

A wide range of methylxanthine molecular targets would make this an appealing field of cancer research via structure-activity approaches [4, 6, 7, 11]. In addition to methylxanthines, xanthine and hypoxanthine might be used to analyze the functional role of the methyl group within methylxanthines for the antitumor functions [11]. In this work, we compared theobromine, theophylline, xanthine, and hypoxanthine with caffeine for the effect on the SRSF3-p53 pathway in HeLa cells [6, 7, 13, 20, 21]. We further identified that theophylline did as well as caffeine and synergistically work together to modulate the alternative splicing function of SRSF3 to alter the status of *p53* isoforms and the cancer progression in human cervical cancer cells and human breast cancer cells. Thus, methylxanthines might have individual structure-activity involved into the development of target therapeutics for various cancers.

## RESULTS

### Theophylline, as well as caffeine, has the ability to switch p53 from an alpha isoform into a beta isoform mediated through the SRSF3-dependent splicing pathway in HeLa cervical cancer cell line

**Figure 1:**
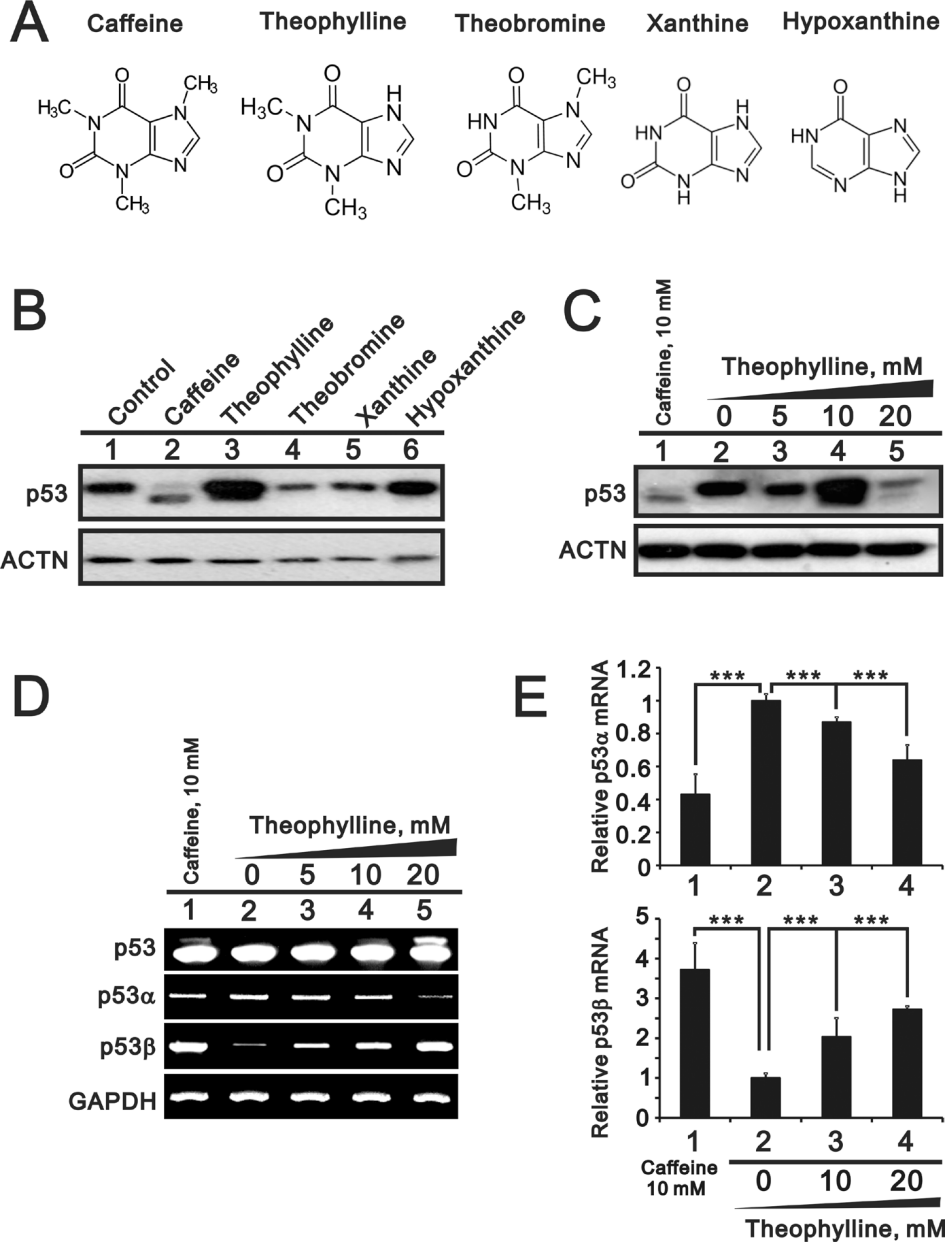
The effects of methylxanthines and xanthines on the alternative splicing of p53 (**A**) The structures of caffeine, theophylline, theobromine, xanthine, and hypoxanthine. (**B**) HeLa cells were treated with 5 mM indicated drugs for 24 h. (**C**) HeLa cells were treated with indicated concentration of theophylline for 24 h. Cell lysates were subject to Western blotting analysis against the p53 antibody. The effect of theophylline on the isoforms of p53 were compared with the effect of caffeine. ACTN was a loading control. (**D** and **E**) HeLa cells were treated with indicated concentration of theophylline for 16 h. (D) Cell lysates were subject to the RT-PCR and GAPDH was a loading control. The results (B–D) are representative of two independent experiments. (E) Cell lysates were subject to the real-time PCR for p53 alpha and beta. The expression amount of p53 alpha or beta was set as the value of one with no methylxanthine treatment. The results are representative of three independent experiments and presented as the mean ± S.D. ^***^P < 0.001.

**Figure 2:**
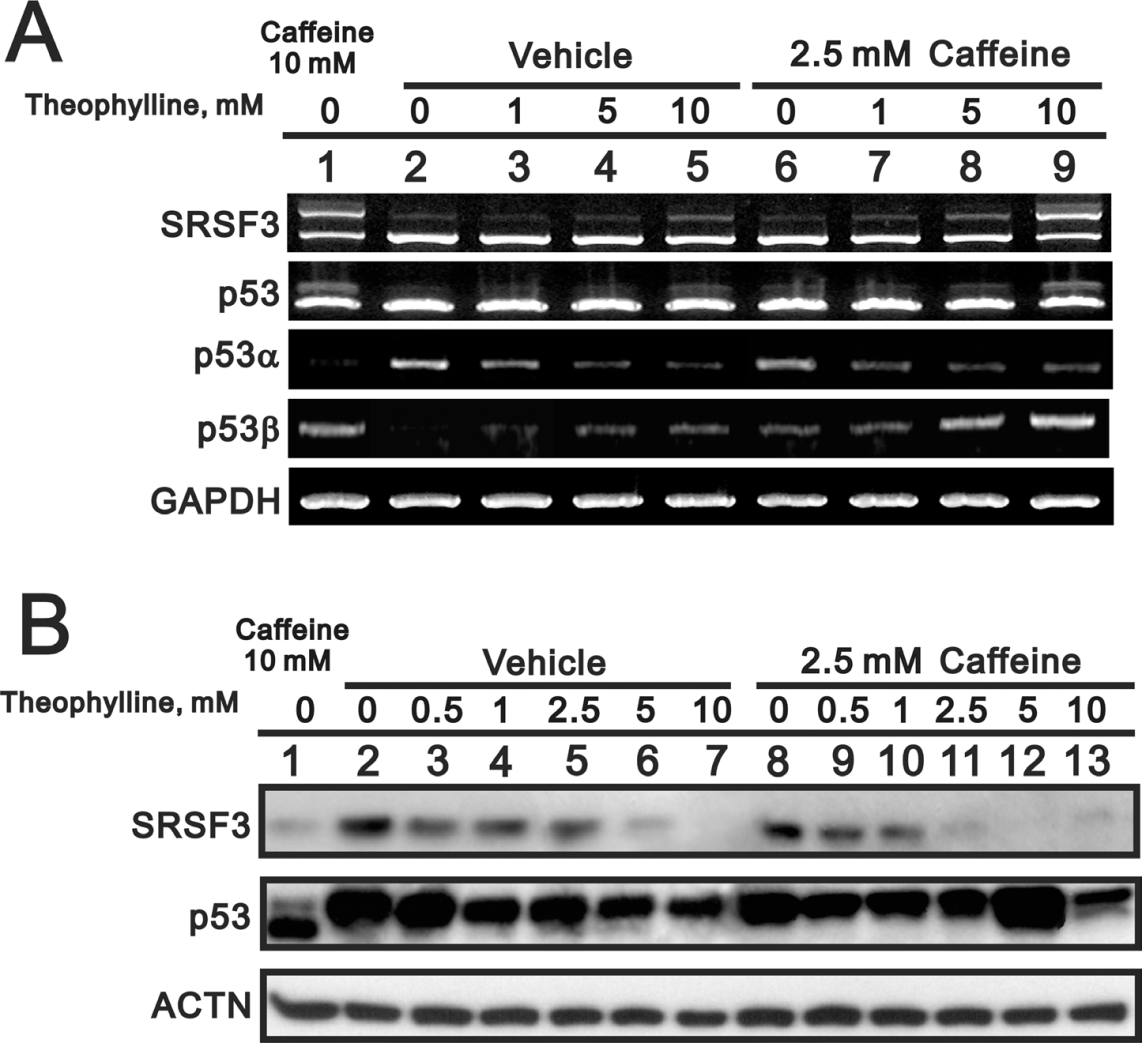
The effect of theophylline on the SRSF3-p53 pathway HeLa cells were treated with indicated concentration of theophylline combined with 2.5 mM caffeine for 24 h. Cell lysates were subject to the RT-PCR (**A**) and Western blotting analysis (**B**). GAPDH was a mRNA loading control and ACTN was a protein loading control. The results are representative of two independent experiments.

Based on many studies of caffeine for antitumor functions [6, 7, 13], we further examined the functional role of other methylxanthines (theophylline and theobromine) and xanthines (xanthine and hypoxanthine) (Figure 1A). Compare to 5 mM caffeine, we chose 5 mM as working concentration of the abovementioned drugs through the screening of the optimal dosage (data not shown). Herein, we used HeLa cells to examine the effects of tested compounds with previous findings of caffeine [7]. We checked the switch of p53α into p53β using Western blotting, RT-PCR, and real-time PCR analysis. Our results showed that only theophylline had the ability to alternatively splice p53 from an α isoform into a β isoform as caffeine did in HeLa cells (Figure 1B–1E). We further confirmed that theophylline, as well as caffeine, switched from p53α into p53β mediated through the downregulation of splicing factor SRSF3 using Western blotting and RT-PCR analysis (Figure 2). Theophylline synergistically acted with caffeine to downregulate the amount of wild-type SRSF3 (Figure 2).

### Theophylline synergistically worked with caffeine to suppress cell survival and colony formation in HeLa cells

**Figure 3:**
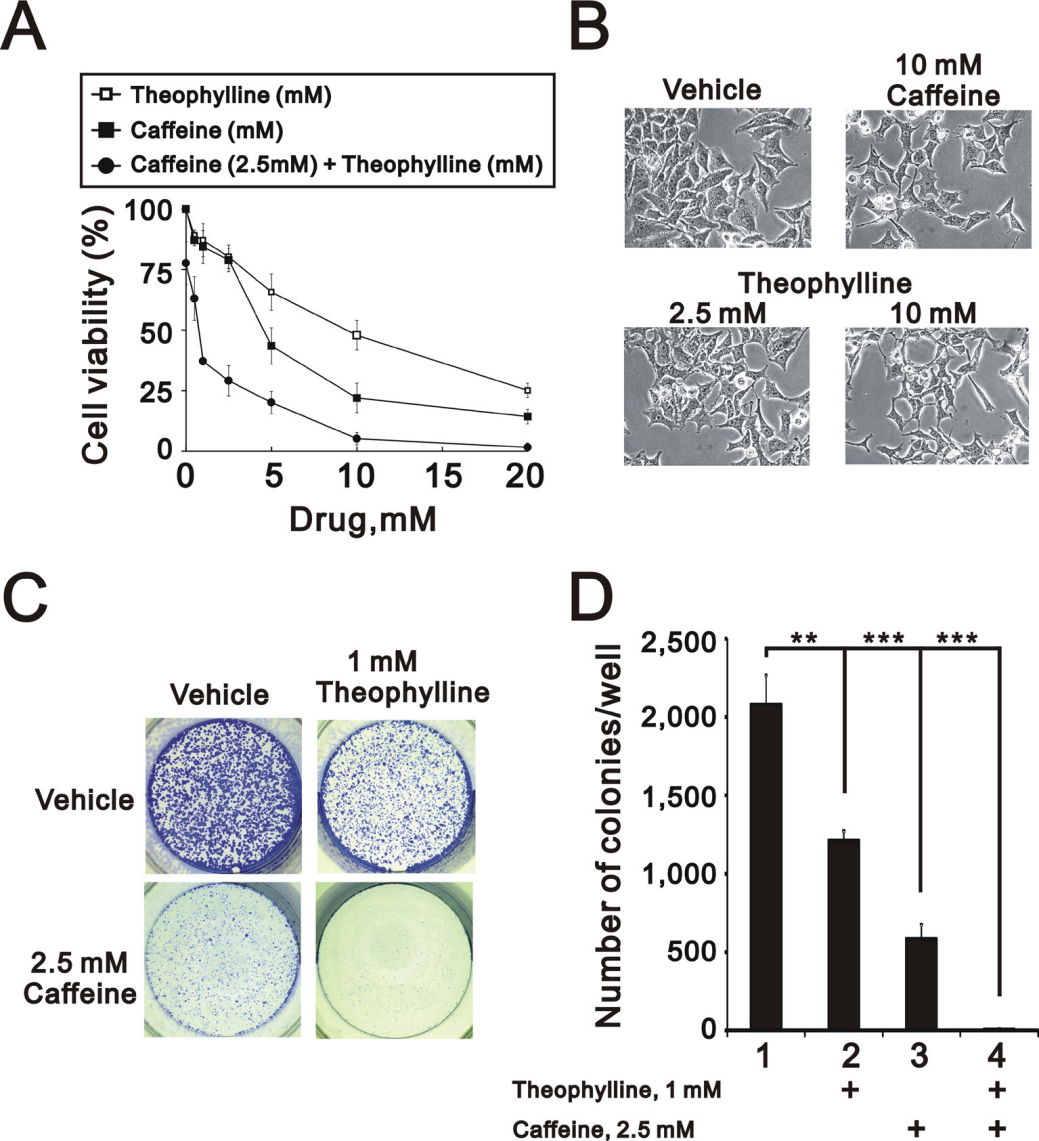
Theophylline synergistically enhanced caffeine-induced cell death (**A**) HeLa cells were treated with indicated amount of theophylline, caffeine, or both combinations for 24 h. Cell viability were measured by MTS assay. (**B**) The morphology of HeLa cells was observed with the indicated amount caffeine or theophylline for 24 h under light microscopy. (**C** and **D**) HeLa cells were treated with indicated amount of theophylline, caffeine, or both combinations for 24 h. The number of colonies was count by methylene blue staining. The results are representative of three independent experiments and presented as the mean ± S.D. ^**^P < 0.01 and ^***^P < 0.001.

Caffeine and theophylline are methylxanthines and have different clinical applications [11, 17]. Figures 1 and 2 suggest that theophylline might have similar antitumor functions to caffeine in HeLa cells. Hence, we further examined the functional roles of theophylline in cell survival and colony formation. Compared to caffeine, theophylline also had the ability to suppress cell survival, induce spindle shape, and colony formation (Figure 3A–3C). In addition, theophylline synergistically worked with caffeine to suppress cell survival and the colony formation in HeLa cells (Figure 3A and 3C), suggesting theophylline had distinctive mechanisms from caffeine.

### Theophylline predominantly suppresses the proliferation rate of HeLa cells

**Figure 4:**
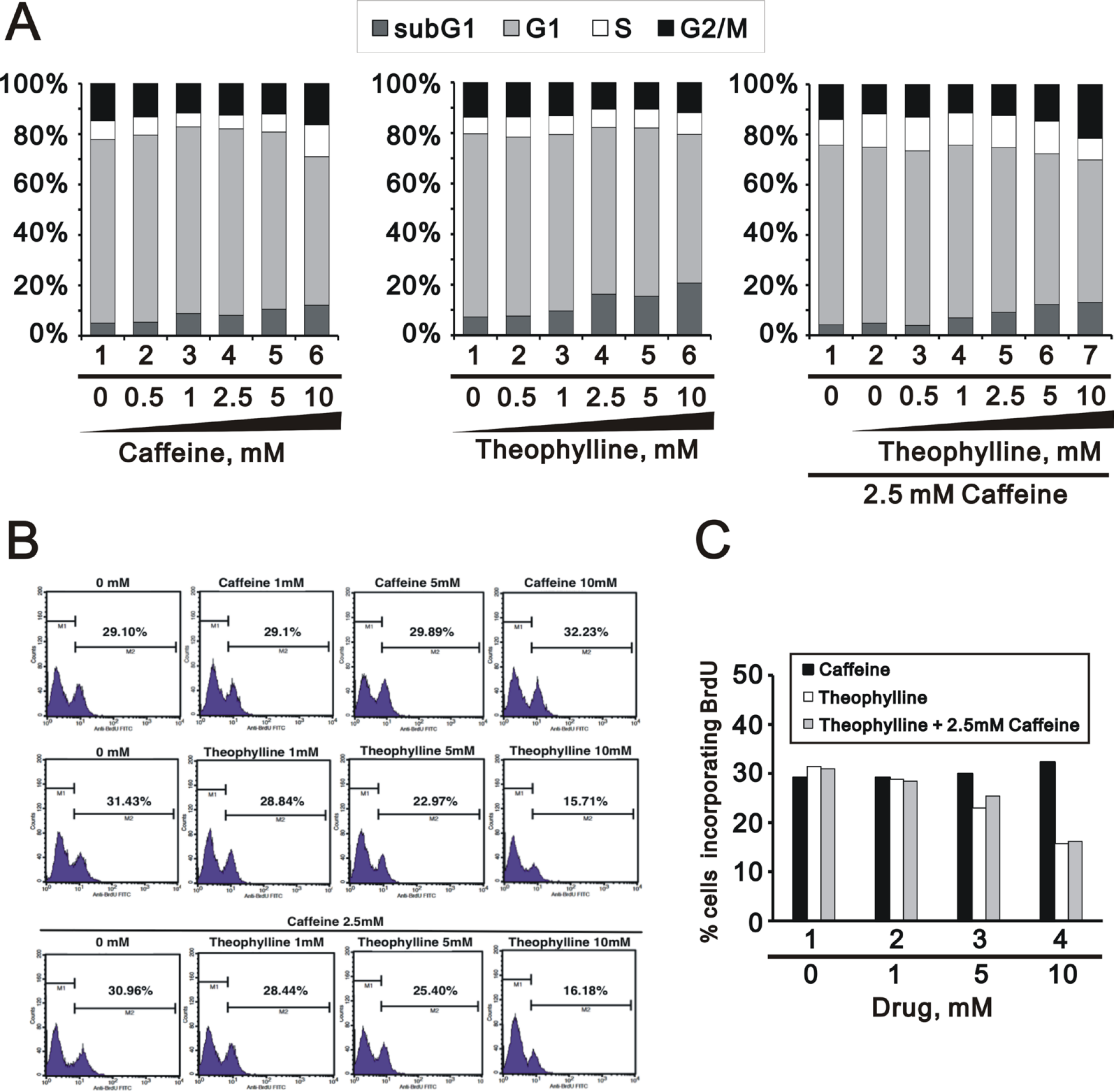
Theophylline suppressed cellular proliferation HeLa cells were treated with indicated amount of theophylline, caffeine or both combinations for 24 h. (**A**) Cell cycle profile were performed with PI dye; (**B** and **C**) Proliferation rate were measured with BrdU using the flow cytometry analysis. The results are representative of three independent experiments.

More functions of theophylline were verified in the cell cycle profile, proliferation rate, and apoptosis. Theophylline, as well as caffeine, induces G2/M arrest via the downregulation of G1 population using flow cytometry analysis in HeLa cells (Figure 4A). Theophylline and caffeine increased the subG1 populations, but no synergistic effect on the subG1 population in the combination of caffeine and theophylline (Figure 4A). Theophylline predominantly suppresses the proliferation rate of HeLa cells using the BrdU proliferation analysis, whereas caffeine had an enhanced effect on the proliferation rate (Figure 4B and 4C). Theophylline and caffeine had no functional interaction on the proliferation rate.

### Theophylline, as well as caffeine, induces apoptosis and switches p53 from an alpha isoform into a beta isoform mediated through the downregulation of SRSF3 in MCF-7 breast cancer cell line

**Figure 5:**
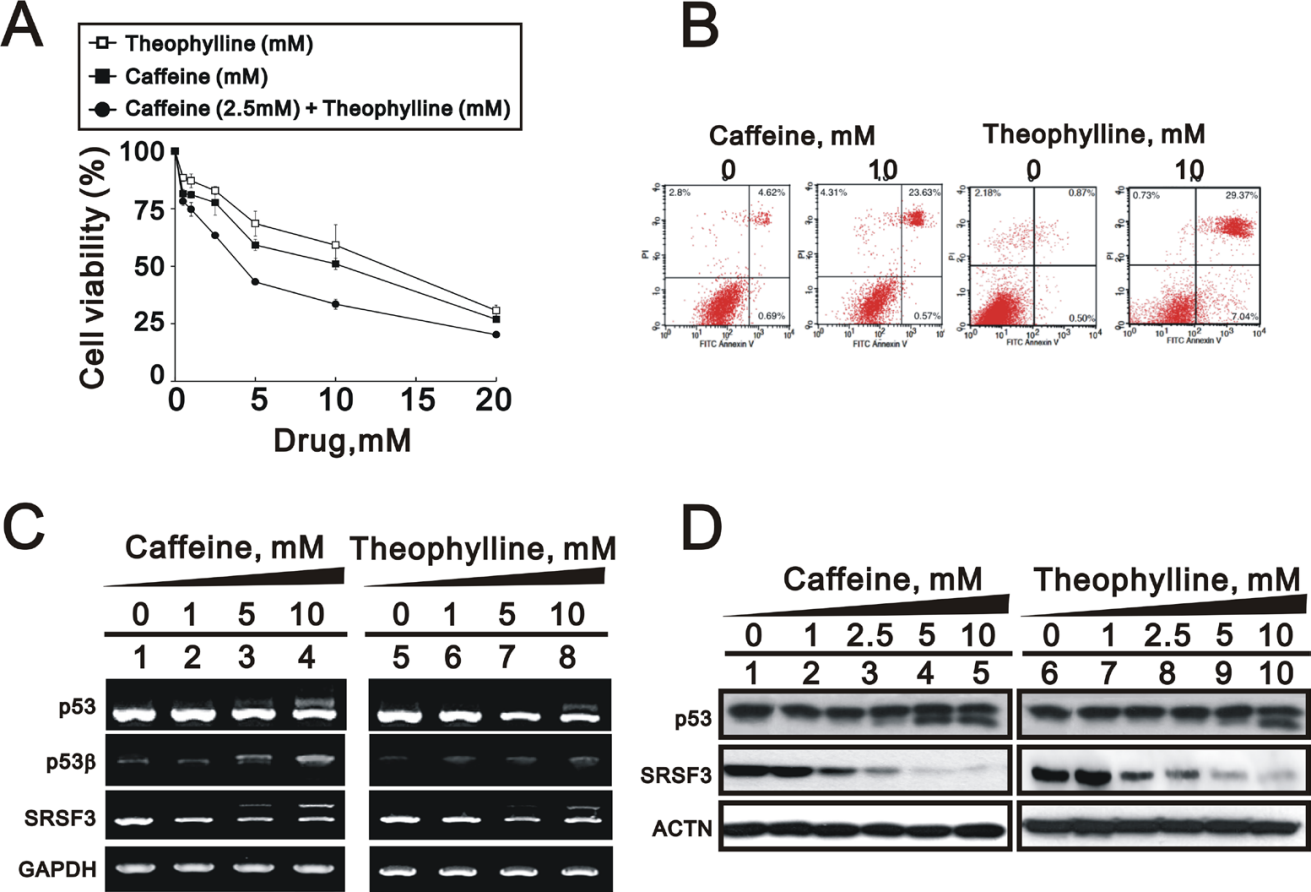
Theophylline induced cell death and apoptosis in MCF-7 cells (**A**) MCF-7 cells were treated with indicated amount of theophylline, caffeine, or both combinations for 24 h. Cell viability were measured with the MTS assay. (**B**) MCF-7 cells were treated with 10 mM theophylline and caffeine for 24 h. The apoptosis analysis was used with Annexin V using the flow cytometry analysis. MCF-7 cells were treated with indicated concentration of theophylline for 24 h. Cell lysates were subject to (**C**) the RT-PCR analysis with primers for p53, p53 beta, SRSF3. GAPDH as a loading control; (**D**) Western blotting analysis with antibodies against p53 and SRSF3. ACTN served as a loading control. The results are representative of two independent experiments.

In addition to human cervical cancer, breast cancer is the most frequent malignancy in females. We further treated one popular breast cancer cell line, MCF-7, with theophylline and caffeine and found that both had the ability to suppress cell survival (Figure 5A). In the flow cytometry with Annexin V and PI, theophylline had effects on early and late apoptosis stages and caffeine only worked on the late apoptosis stage (Figure 5B). Using Western blotting and RT-PCR analysis, we found that theophylline and caffeine had the ability to switch p53 from an alpha isoform into a beta isoform mediated through the downregulation of SRSF3 in MCF-7 cells (Figure 5C and 5D).

### Theophylline, as well as caffeine, induces G2/M arrest via the downregulation of G1 population in MCF-7 cells

**Figure 6:**
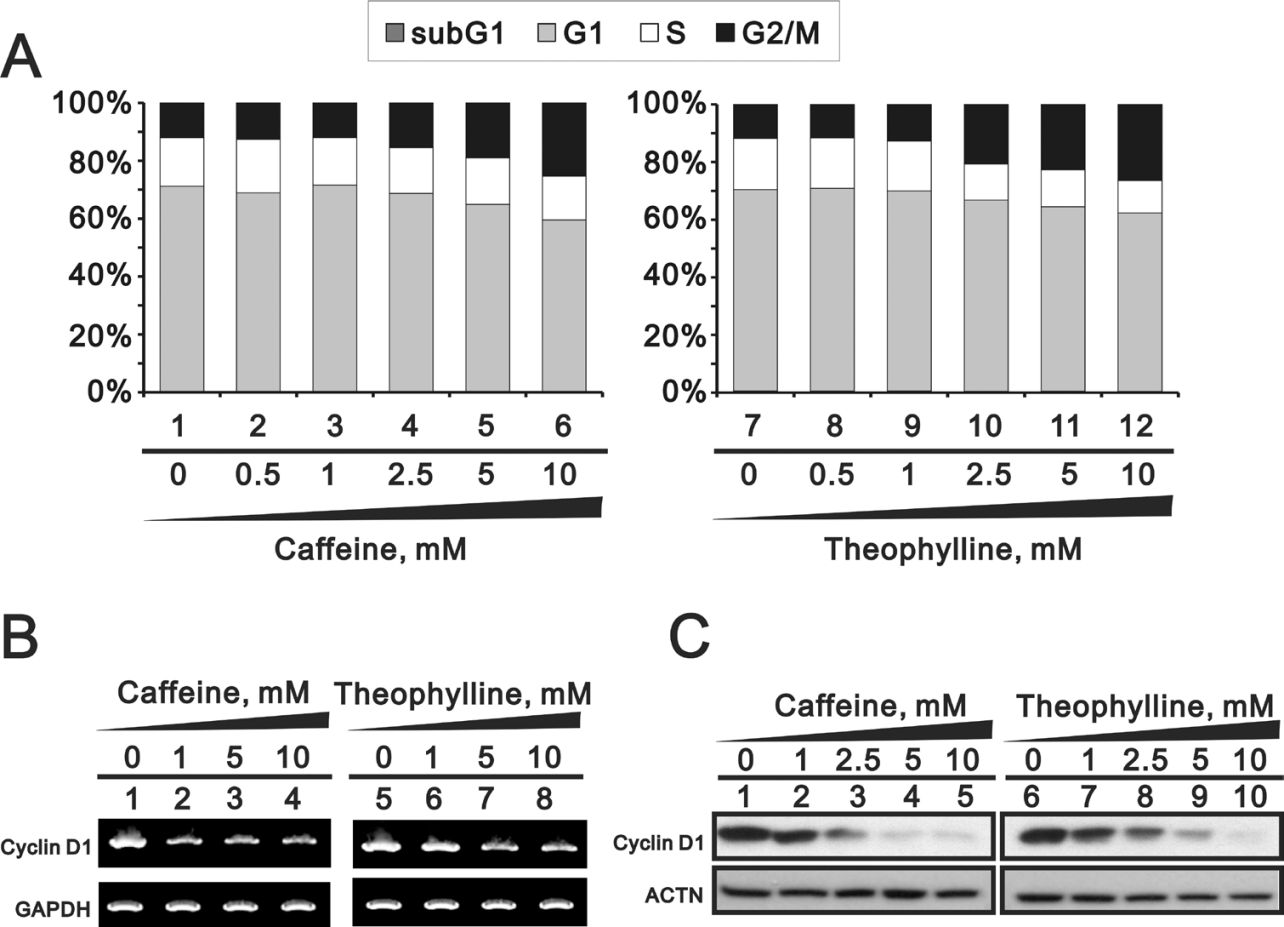
Theophylline and caffeine caused the G2/M arrest MCF-7 cells were treated with indicated amount of theophylline and caffeine for 24 h. (**A**) Cell cycle profile were performed with PI dye using the flow cytometry analysis. Cell lysates were subject to (**B**) the RT-PCR analysis with primers for cyclin D1. GAPDH was used as a loading control; (**C**) Western blotting analysis with antibodies against cyclin D1. ACTN served as a loading control. The results are representative of two independent experiments

**Figure 7:**
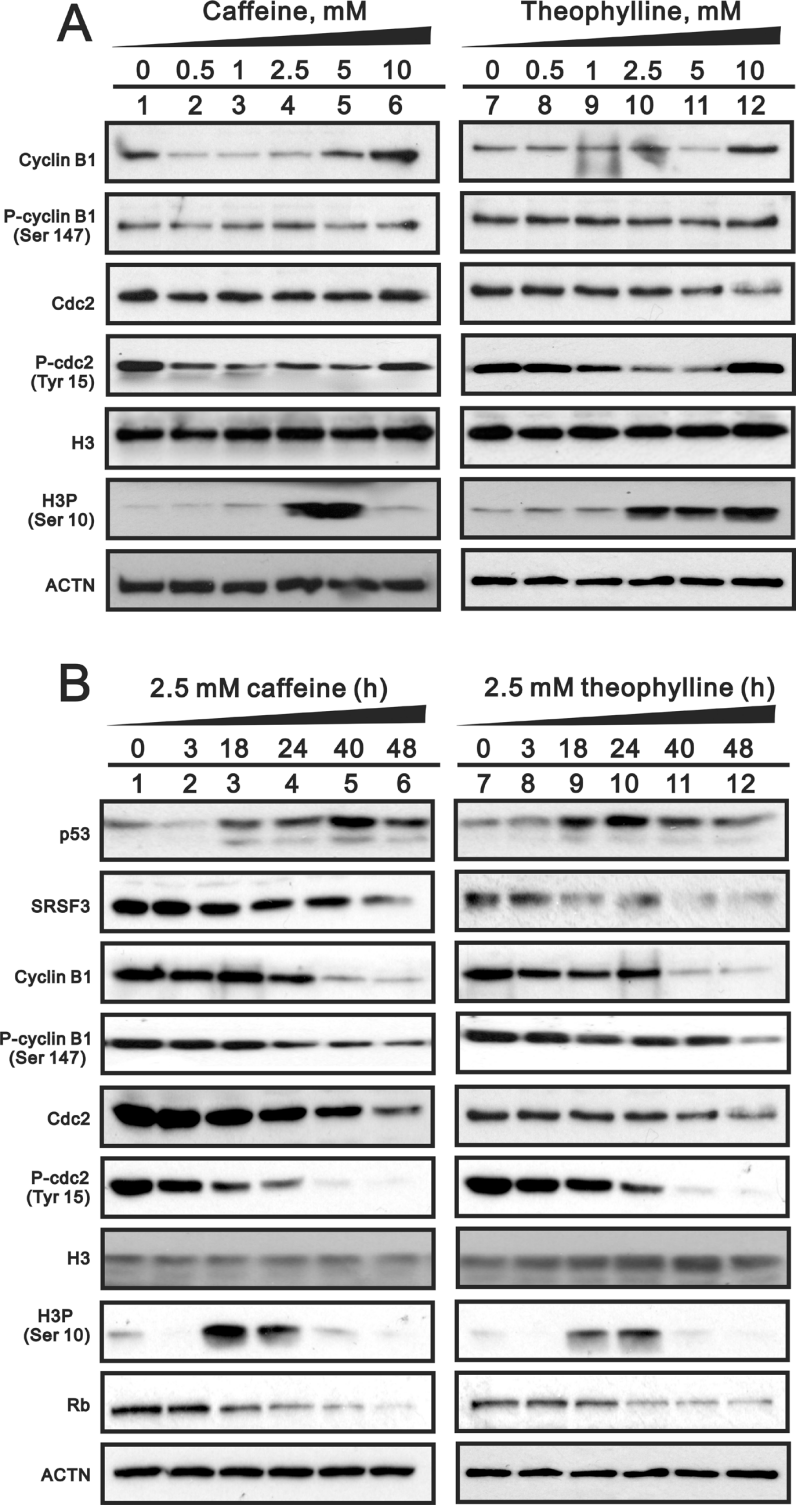
The dose- and time-course effects of theophylline and caffeine on selected protein expression. (A) MCF-7 cells were treated with indicated amount of theophylline and caffeine for 24 h. **(B)** MCF-7 cells were treated with 2.5 mM theophylline and caffeine for indicated time. Cell lysates were subject to Western blotting analysis with antibodies against indicated proteins. ACTN was a loading control. The results are representative of two independent experiments.

Theophylline, as well as caffeine, induced G2/M arrest mediated through the downregulation of G1 population using flow cytometry analysis in MCF-7 cells (Figure 6A). The downregulation of G1 population was verified to be mediated through the decrease of cyclin D1 gene and protein expression using Western blotting and RT-PCR analysis (Figure 6B and 6C). We further investigated the mechanism of G2/M arrest and found that the induction of G2/M marker H3P (serine 10 phosphorylation on Histone H3) was followed a caffeine or theophylline dose- and time-dependent manner (Figure 7). The phosphorylation of specific residues of Cyclin B1 (serine 147) and Cdc2 (tyrosine 15) were verified for the G2/M arrest in MCF-7 cells.

### Theophylline, as well as caffeine, induce epithelial-to-mesenchymal transition (EMT) and senescence and suppress colony formation in MCF-7 cells

**Figure 8:**
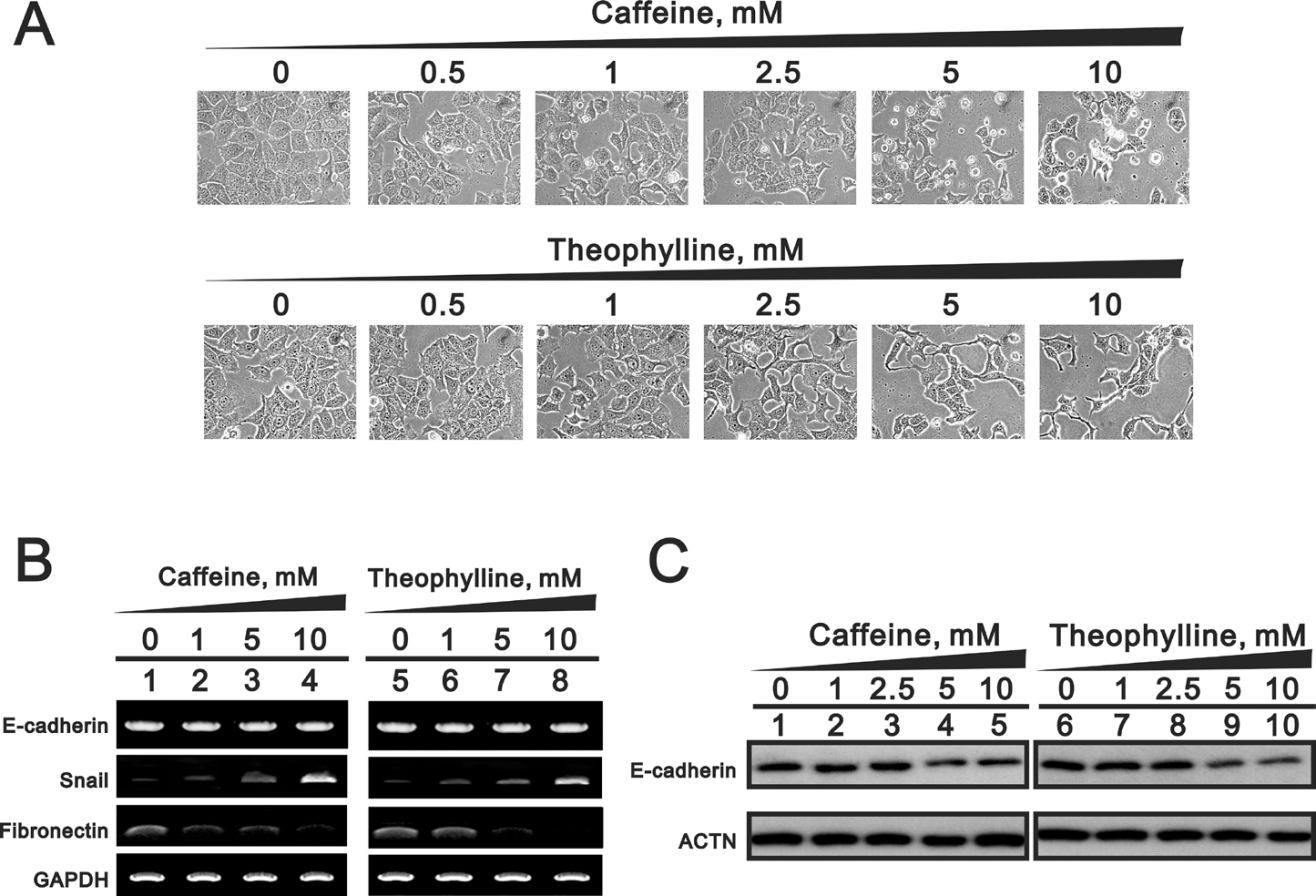
The EMT effects by theophylline and caffeine in MCF-7 cells MCF-7 cells were treated with indicated amount of theophylline and caffeine for 24 h. (**A**) The cell morphology was observed using light microscopy. Cell lysates were subject to (**B**) the RT-PCR analysis with primers for E-cadherin, snail, and fibronectin. GAPDH served as a loading control; (**C**) Western blotting analysis with antibodies against E-cadherin. ACTN was used as a loading control. The results are representative of three independent experiments.

Some genes are involved in EMT and are also alternatively spliced by SRSF3, which could be modulated by caffeine [7]. We observed the morphology of theophylline- or caffeine-treated MCF-7 cells from round shape into spike-like types and implied that the induction of EMT by theophylline and caffeine (Figure 8A). Our RT-PCR data revealed that the mesenchymal marker *Snail* mRNA was induced and the epithelial marker *fibronectin* mRNA was repressed (Figure 8B). The epithelial marker *E-cadherin* gene remained constant, whereas E-cadherin protein was suppressed using the RT-PCR and Western blotting analysis (Figure 8B and 8C).

**Figure 9:**
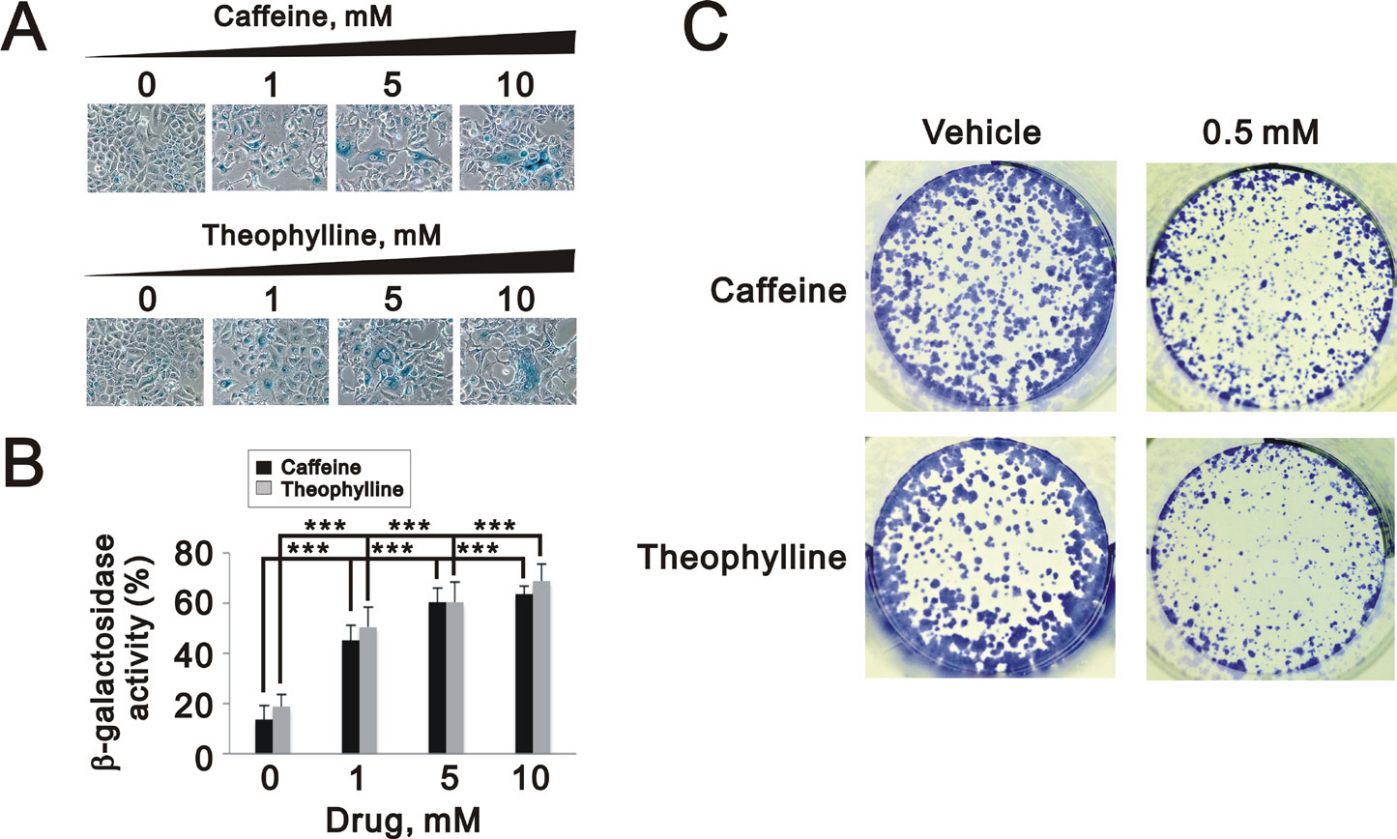
Theophylline and caffeine caused cellular senescence in MCF-7 cells (**A** and **B**) MCF-7 cells were treated with indicated amount of theophylline and caffeine for 24 h. The senescence activity was measured and quantified with SA β-galactosidase analysis. The results are representative of three independent experiments and presented as the mean ± S.D. ^***^P < 0.001. (**C**) MCF-7 cells were treated with 0.5 mM caffeine and theophylline and for the colony formation analysis. The results are representative of two independent experiments.

The quantitative data suggested that theophylline and caffeine had the ability to significantly induce cellular senescence and suppress the colony formation in MCF-7 cells, respectively (Figure 9A and 9C).

### Theophylline and caffeine fail to modulate cell morphology, p53, and SRSF3 in the normal breast epithelial cells

**Figure 10:**
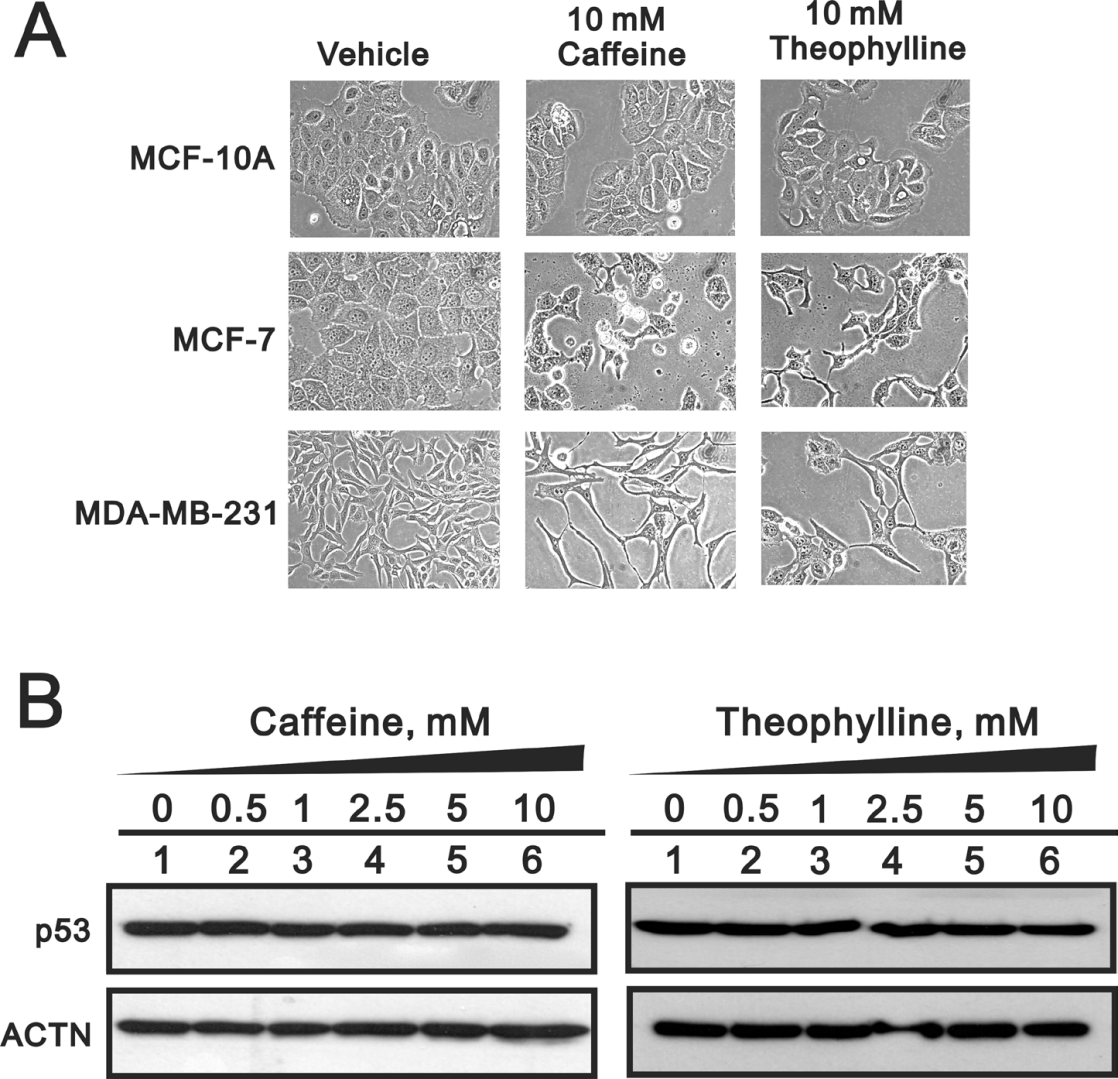
Theophylline had no effect on normal breast cells (**A**) MCF-10A, MCF-7 and MDA-MB-231 cells were treated with 10 mM theophylline and caffeine for 24 h. The cell morphology was observed using light microscopy. (**B**) MCF-10A cells were treated with indicated amount of theophylline and caffeine for 24 h. Cell lysates were subject to Western blotting analysis with antibodies against p53. ACTN was a loading control. The results are representative of two independent experiments.

To test the specificity of theophylline and caffeine as antitumor drugs for breast cancers, we further examined the effects of theophylline and caffeine in one normal breast cell line and one triple negative breast cancer cell line, MCF-10A and MDA-MB-231 cells, respectively. We failed to observe the morphology change in MCF-10A, compared with the spindle shapes in MCF-7 and MDA-MB-231 cells (Figure 10A). In addition, the p53 protein remained constant level in theophylline- or caffeine-treated MCF-10A cells using Western blotting analysis (Figure 10B). Our data suggests that theophylline and caffeine might specifically suppress breast tumor cells, not normal cells, for their antitumor functions.

## DISCUSSION

In this report, we first describe SRSF3 as a novel target of theophylline against breast and cervical cancer cell growth. Specifically, we demonstrate that theophylline can increase p53β by alternative splicing of p53 pre-mRNA through suppressing SRSF3. Theophylline can synergistically enhance the caffeine-induced cell death and suppress cell proliferation and colony formation, as well as cell apoptosis. In terms of specificity of theophylline in cancer therapy, we show that only malignant breast cancer cell MCF-7 and MDA-MB-231 are vulnerable to theophylline and caffeine treatment, whereas normal breast epithelial cell MCF-10A remains unaffected.

SRSF3 is the first case in which it modifies the splicing of its own mRNA and SRSF1 antagonizes this modulation [22]. SRSF3 has been shown to regulate the splicing of abovementioned genes, including *p53* [7–10]. Knockout studies indicated that SRSF3 is essential for mouse development, hepatocyte differentiation, and metabolic function, as well as tumor cell proliferation and maintenance [9, 23–25]. The downregulation of *SRSF3* gene expression might have the ability to regulate tumor initiation, progression, and maintenance [23, 24]. Recent study demonstrated that SRSF3 induces cellular senescence via the *p53* alternative splicing to switch alpha isoform into beta isoform. The latest finding demonstrated that human *SRSF3* gene generates two alternative splice transcripts, encoding a full-length protein and a truncated isoform which is degraded through the nonsense-mediated mRNA decay pathway [26]. Hence, the detailed regulatory mechanisms of methylxanthines for human *SRSF3* gene expression remain to be addressed.

Despite the alternative splicing of genes via SRSF3, theophylline also modulated cellular apoptosis, senescence, and colony formation. However, theophylline had a distinctive effect on the cellular proliferation from caffeine [11, 16, 17]. These findings revealed that theophylline had the similar functions as caffeine and possessed some unique functional roles, such as the suppression of cellular proliferation rate. Summarily, theophylline might perform its antitumor functions via the working mechanisms of SRSF3 splicing factor, methylxanthines and unique molecules, such NF-κB inhibition, histone deacetylase 2 activity augmentation and interleukine-10 secretion [11, 16, 17]. It remains to be investigated how theophylline suppress cell proliferation and what are the potential candidate target(s) modulated by theophylline. Nevertheless, our work repurposes theophylline as an antitumor drug like caffeine.

Caffeine, theophylline and theobromine are three most relevant methylxanthines which are produced from botanical species and can be found in daily beverages and foods, such as coffee, tea, and chocolate. Caffeine and theophylline are present in coffee, tea, energetic beverages, and chocolate. Theobromine is discovered in tea and chocolate, but is also a caffeine metabolite in the body [27]. In plants, methylxanthines are formed from purine nucleotides and can also be obtained by chemical synthesis. In this study, we purchased three abovementioned compounds via the chemical synthesis. The therapeutic serum concentration of theophylline for mild persistent asthma patient is between 5 and 15 μg/mL (27.8 and 83.8 μM) [28]. Herein, we can only observe the splicing effect of theophylline on p53 at the concentration over 5 mM, which is much higher than therapeutic serum concentration. Although the cell viability of the benign breast epithelial cell MCF-10A remained unaffected at 10 mM of theophylline, it is still not feasible for systematic treatment at millimolar range. Despite the lack of efficacy to reach minimum safety dosage in our study, theophylline or other methylxanthine derivatives can be a promising lead compound for targeting SRSF3 in anti-cancer drug development.

From a structure-activity relationship perspective, we verified the functional importance of the three methyl groups on the xanthine backbone of caffeine (1,3,7-trimethylxanthine) by comparing with two other methylxanthines, theophylline (1,3-dimethylxanthine) and theobromine (3,7-dimethylxanthine) in this study. Our findings revealed that theophylline, but not theobromine, has a similar ability to alternatively splice the pre-mRNA of p53 via the downregulation of *SRSF3*, which is similar to caffeine in our previous work [7]. Our results imply that the methyl groups at positions 1 and 3 of methylxanthine derivatives are important for pharmacological targeting of *SRSF3*. Intriguingly, two research articles using methylxanthine-derived compounds as anticancer agents also retain the methyl groups at position 1 and 3 [29, 30], which is consistent with our results. It will be interesting to evaluate their effects on SRSF3. In contrast to theobromine, theophylline is a mid-product of caffeine slowly degraded by consecutive removal of the three methyl groups in a plant [11, 17]. The structure-activity relationships for methylxanthines in their antagonism of adenosine have been demonstrated and the 1-methyl group in the methylxanthines is pivotal for its inhibitory effects exerted at the adenosine receptor level [11, 17, 31]. Our findings also revealed the 1-methyl group might be important for antitumor functions of methylxanthines based on the response of theobromine. In clinical applications, caffeine primarily stimulates the central nervous system and respiratory system and theophylline is more effective than caffeine in cardiac stimulation, coronary dilatation, and smooth muscle relaxation [12, 32–39]. Theobromine is generally less active than caffeine or theophylline [11, 17]. In addition to these direct anti-tumor effects, methylxanthines have been administrated along other conventional cancer treatments by promotion of arrest or abrogation of the cell cycle checkpoints, namely the G2/M checkpoint [6, 7, 13–15].

Different types of cell death are often defined by morphological criteria [40] and theophylline and caffeine had dramatic effects on the cell morphologies in HeLa and MCF-7 cells. Compare to the apoptotic effect of theophylline and caffeine on HeLa cells (Figure 4A), theophylline and caffeine failed to increase subG1 population in MCF-7 cells. Although we observed slightly increased early apoptotic cells in MCF-7 treated with theophylline, but not caffeine, the possibility of other cell death mechanisms cannot be excluded [7]. Moreover, caffeine and theophylline markedly induced cellular senescence (Figure 9A and 9B) implying the observation of significant G2/M arrest (Figure 6A) could be a permanent event. Heterogeneity in a single cell line may explain why theophylline can induce cell death in some subpopulation, but also cause cellular senescence in other subpopulation of MCF-7 cells [41].

In summary, our findings support that theophylline as well as caffeine had potential abilities to serve as a direct antitumor drug mediated through, at least in part, the SRSF3-p53 pathway and well-known functional roles of methylxanthines. This work provides the reposition of theophylline to develop combinatory therapy for various cancers.

## MATERIALS AND METHODS

### Cell culture and reagents

HeLa, MCF-7, MCF-10A, and MDA-MB-231 cells were cultured in Dulbecco's modified Eagle's medium supplemented with 10% fetal bovine serum and 1% penicillin-streptomycin (Invitrogen, USA). Methylxanthines (caffeine, theophylline, and theobromine) and xanthines (xanthine and hypoxanthine) were purchased from Sigma-Aldrich (USA).

### Western blot analysis

Cell lysates were prepared in lysis buffer (100 mM Tris-HCl of pH 8.0, 150 mM NaCl, 0.1% SDS, and 1% Triton X-100) at 4°C. The cell extracts were separated by SDS-PAGE, transferred onto a polyvinylidene difluoride membrane (Millipore, USA) and detected using antibodies against α-actinin (ACTN), p53, p21, SRSF3, cyclin D1 (Santa Cruz Biotechnology, USA), cyclin B1, p-cyclin B1 (phosphorylation at Ser 147), cdc2, p-cdc2 (phosphorylation at Tyr 15), Histone H3, p-Histone H3 (phosphorylation at Ser 10, H3P) (Cell Signaling, USA), and E-cadherin (BD, USA).

### Reverse transcription-polymerase chain reaction (RT-PCR)

Total RNA was isolated using the TRIsure (BIOLINE, UK) reagent according to the manufacturer's instructions. One microgram of total RNA was subjected to reverse transcription using MMLV reverse transcriptase (Epicentre Biotechnologies, USA) for 60 min at 37°C. The PCR reactions were performed on a GeneAmp PCR system 9700 (Applied Biosystems, USA). All PCR primer sequences are listed in Table 1.

**Table 1:** PCR primers used in this study

Gene name	Primer sequence (5′→3′)
***cyclin D1***	Forward: 5′-ATGGAACACCAGCTCC-3′
	Reverse: 5′-TCAGATGTCCACGTCCCGC-3′
***E-cadherin***	Forward: 5′-CCTGGGACTCCACCTACAGA-3′
	Reverse: 5′-GGATGACACAGCGTGAGAGA-3′
***fibronectin***	Forward: 5′-CAGTGGGAGACCTCGAGAAG-3′
	Reverse: 5′-TCCCTCGGAACATCAGAAAC-3′
***GAPDH***	Forward: 5′-CTTCATTGACCTCAACTAC-3′
	Reverse: 5′-GCCATCCACAGTCTTCTG-3′
***p53***	Forward: 5′-CTCTGACTGTACCACCATCCACTA-3′
	Reverse: 5′-GAGTTCCAAGGCCTCATTCAGCTC-3′
***p53α***	Forward: 5′-GATGAAGCTCCCAGAATGCCAGAG-3′
	Reverse: 5′-GAGTTCCAAGGCCTCATTCAGCTC-3′
***p53β***	Forward: 5′-ATGGAGGAGCCGCAGTCAGAT-3′
	Reverse: 5′-TTTGAAAGCTGGTCTGGTC-3′
***SRSF3***	Forward: 5′-ATGCATCGTGATTCCTGTCCATTG-3′
	Reverse: 5′-CTATTTCCTTTCATTTGACCTAGATC-3′
***Snail***	Forward: 5′-ATGCCGCGCTCTTTCCTCGTCAGG-3′
	Reverse: 5′-TCAGCGGGGACATCCTGAGCAGCC-3′

### Cell survival analysis

MTS assay, cells were seeded in 96-well culture plates and incubated for the time periods indicated. The MTS assay reagent consists of MTS and the electron coupling agent phenazine methosulphate (PMS). The 400 μl MTS/PMS solution was added to each well and the plates were incubated for 3 h at 37°C. Transfer 100 μl aliquots of each sample and the absorbance at 490 nm was measured using an ELISA plate reader (Multiskan EX, Thermo, USA). As a control, cells treated only with media containing no compounds were considered as 100% cell survival.

### Clonogenic survival assay

HeLa and MCF7 cells were seeded into 6-well plates and treated with the designated concentrations of caffeine 24 h after seeding. The cells were fed at an interval of 3 days with fresh medium for two weeks. Subsequently, the cells were fixed and stained with 2% methylene blue (Sigma) in 50% methanol for 5 min.

### Fluorescence-activated cell sorting (FACS), cell cycle profile, proliferation and apoptosis analysis

For cell cycle analysis, the distribution was determined by measuring DNA content using FACS. The cells were fixed in 70% ice-cold ethanol and kept at -20°C overnight. Before analysis, the harvested cells were washed with ice-cold phosphate buffered saline, PBS, twice and stained with propidium iodide (PI) solution (5 μg/ml PI in PBS, 0.5% Triton x-100, and 0.5 μg/ml RNase A) for 30 min at 37°C in the dark. For the proliferation analysis, the cells were treated and then processed with the FITC-BrdU (5-bromo-2-deoxyuridine) Flow Kits according to the manufacturer's instructions (BD Biosciences). All the samples were analyzed by the FACSCalibur flow cytometer (BD Biosciences). Data was analyzed by the Cell Quest Pro software (BD Biosciences). For early and late apoptosis analysis, the cells were measured by PE Annexin V Apoptosis Detection Kit (BD Biosciences) and APO-DIRECT Kit (BD Biosciences), respectively. The cells were treated and then processed according to the manufacturer's instructions.

### *In situ* staining for senescence-associated (SA) β-galactosidase activity

Cultured cells were washed in PBS (pH 7.4), incubated in a solution of 2% formaldehyde/0.2% glutaraldehyde, and then incubated overnight at 37°C in freshly prepared staining solution consisting of 1 mg/mL X-gal (5-bromo-4-chloro-3-indolyl-β-D-galactoside), 5 mM K_3_Fe(CN)_6_, 5 mM K_4_Fe(CN)_6_, 150 mM NaCl, and 2 mM MgCl_2_ in 40mM citric acid/sodium phosphate, pH 6.0. Following this incubation, cells were washed with water and examined by light microscopy at 200× magnification.

### Statistical analysis

Statistical values are expressed as the means ± SD of at least three independent experiments. All comparisons between groups were performed using the unpaired two-tailed *t*-test. Statistical significance was set at *p <* 0.05.

## Abbreviations

SRSFs: serine/arginine-rich splicing factors
KLF6: Krüppel-like factor 6
HIF-1α: *hypoxia-inducible* factor-1α
NF-κB: nuclear factor-kappa B
ACTN: β-actinin
H3P: p-Histone H3 (phosphorylation at Ser 10)
RT-PCR: Reverse transcription-polymerase chain reaction
MTS: 3-(4,5-dimethylthiazol-2-yl)-5-(3-carboxymethoxyphenyl)-2-(4-sulfophenyl)-2H-tetrazolium
PMS: phenazine methosulphate
FACS: fluorescence-activated cell sorting
PI: propidium iodide
PBS: phosphate buffered saline
BrdU: 5-bromo-2-deoxyuridine
SA: senescence-associated
EMT: epithelial-to-mesenchymal transition

## References

[1] Erkelenz S, Mueller WF, Evans MS, Busch A, Schöneweis K, Hertel KJ, Schaal H (2013). Position-dependent splicing activation and repression by SR and hnRNP proteins rely on common mechanisms. RNA.

[2] Busch A, Hertel KJ (2012). Evolution of SR protein and hnRNP splicing regulatory factors. Wiley Interdiscip Rev RNA.

[3] Graveley BR (2000). Sorting out the complexity of SR protein functions. RNA.

[4] Anczuków O, Krainer AR (2016). Splicing-factor alterations in cancers. RNA.

[5] Anko ML, Muller-McNicoll M, Brandl H, Curk T, Gorup C, Henry I, Ule J, Neugebauer KM (2012). The RNA-binding landscapes of two SR proteins reveal unique functions and binding to diverse RNA classes. Genome Biology.

[6] Shi J, Hu Z, Pabon K, Scotto KW (2008). Caffeine regulates alternative splicing in a subset of cancer-associated genes: a role for SC35. Mol Cell Biol.

[7] Lu GY, Huang SM, Liu ST, Liu PY, Chou WY, Lin WS (2014). Caffeine induces tumor cytotoxicity via the regulation of alternative splicing in subsets of cancer-associated genes. Int J Biochem Cell Biol.

[8] Walsh CM, Suchanek AL, Cyphert TJ, Kohan AB, Szeszel-Fedorowicz W, Salati LM (2013). Serine arginine splicing factor 3 is involved in enhanced splicing of glucose-6-phosphate dehydrogenase RNA in response to nutrients and hormones in liver. J Biol Chem.

[9] Sen S, Jumaa H, Webster NJ (2013). Splicing factor SRSF3 is crucial for hepatocyte differentiation and metabolic function. Nat Commun.

[10] Biamonti G, Bonomi S, Gallo S, Ghigna C (2012). Making alternative splicing decisions during epithelial-to-mesenchymal transition (EMT). Cell Mol Life Sci.

[11] Monteiro JP, Alves MG, Oliveira PF, Silva BM (2016). Structure-Bioactivity Relationships of Methylxanthines: Trying to Make Sense of All the Promises and the Drawbacks. Molecules.

[12] Bode AM, Dong Z (2007). The enigmatic effects of caffeine in cell cycle and cancer. Cancer Lett.

[13] Ito K, Nakazato T, Miyakawa Y, Yamato K, Ikeda Y, Kizaki M (2003). Caffeine induces G2/M arrest and apoptosis via a novel p53-dependent pathway in NB4 promyelocytic leukemia cells. J Cell Physiol.

[14] Hirsh L, Dantes A, Suh BS, Yoshida Y, Hosokawa K, Tajima K, Kotsuji F, Merimsky O, Amsterdam A (2004). Phosphodiesterase inhibitors as anti-cancer drugs. Biochem Pharmacol.

[15] Sugimoto N, Miwa S, Hitomi Y, Nakamura H, Tsuchiya H, Yachie A (2014). Theobromine, the primary methylxanthine found in Theobroma cacao, prevents malignant glioblastoma proliferation by negatively regulating phosphodiesterase-4, extracellular signal-regulated kinase, Akt/mammalian target of rapamycin kinase, and nuclear factor-kappa B. Nutr Cancer.

[16] Gomaa A, Elshenawy M, Afifi N, Mohammed E, Thabit R (2009). Enhancement of the anti-inflammatory and anti-arthritic effects of theophylline by a low dose of a nitric oxide donor or non-specific nitric oxide synthase inhibitor. Br J Pharmacol.

[17] Theophylline Barnes PJ (2013). Am J Respir Crit Care Med.

[18] Papadimitriou A, Silva KC, Peixoto EB, Borges CM, Lopes de Faria JM, Lopes de Faria JB (2015). Theobromine increases NAD+/Sirt-1 activity and protects the kidney under diabetic conditions. Am J Physiol Renal Physiol.

[19] Baggott MJ, Childs E, Hart AB, de Bruin E, Palmer AA, Wilkinson JE, de Wit H (2013). Psychopharmacology of theobromine in healthy volunteers. Psychopharmacology (Berl).

[20] Zuckerman V, Wolyniec K, Sionov RV, Haupt S, Haupt Y (2009). Tumour suppression by p53: the importance of apoptosis and cellular senescence. J Pathol.

[21] Marcel V, Dichtel-Danjoy ML, Sagne C, Hafsi H, Ma D, Ortiz-Cuaran S, Olivier M, Hall J, Mollereau B, Hainaut P, Bourdon JC (2011). Biological functions of p53 isoforms through evolution: lessons from animal and cellular models. Cell Death Differ.

[22] Jumaa H, Nielsen PJ (1997). The splicing factor SRp20 modifies splicing of its own mRNA and ASF/SF2 antagonizes this regulation. EMBO J.

[23] He X, Arslan AD, Pool MD, Ho TT, Darcy KM, Coon JS, Beck WT (2011). Knockdown of splicing factor SRp20 causes apoptosis in ovarian cancer cells and its expression is associated with malignancy of epithelial ovarian cancer. Oncogene.

[24] Jia R, Li C, McCoy JP, Deng CX, Zheng ZM (2010). SRp20 is a proto-oncogene critical for cell proliferation and tumor induction and maintenance. Int J Biol Sci.

[25] Jumaa H, Wei G, Nielsen PJ (1999). Blastocyst formation is blocked in mouse embryos lacking the splicing factor SRp20. Curr Biol.

[26] Kano S, Nishida K, Kurebe H, Nishiyama C, Kita K, Akaike Y, Kajita K, Kurokawa K, Masuda K, Kuwano Y, Tanahashi T, Rokutan K (2014). Oxidative stress-inducible truncated serine/arginine-rich splicing factor 3 regulates interleukin-8 production in human colon cancer cells. Am J Physiol Cell Physiol.

[27] Stavric B (1988). Methylxanthines: toxicity to humans 1. Theophylline. Food Chem Toxicol.

[28] National Asthma Education and Prevention Program (2007). Expert Panel Report 3 (EPR-3): Guidelines for the Diagnosis and Management of Asthma-Summary Report 2007. J Allergy Clin Immunol.

[29] Lentini A, Tabolacci C, Nardi A, Mattioli P, Provenzano B, Beninati S (2012). Preclinical evaluation of the antineoplastic efficacy of 7-(2-hydroxyethyl)theophylline on melanoma cancer cells. Melanoma Res.

[30] Ruddarraju RR, Murugulla AC, Kotla R, Chandra Babu Tirumalasetty M, Wudayagiri R, Donthabakthuni S, Maroju R, Baburao K, Parasa LS (2016). Design, synthesis, anticancer, antimicrobial activities and molecular docking studies of theophylline containing acetylenes and theophylline containing 1,2,3-triazoles with variant nucleoside derivatives. Eur J Med Chem.

[31] Baraldi PG, Cacciari B, Romagnoli R, Merighi S, Varani K, Borea PA, Spalluto G (2000). A(3) adenosine receptor ligands: history and perspectives. Med Res Rev.

[32] Han W, Ming M, He YY (2011). Caffeine promotes ultraviolet B-induced apoptosis in human keratinocytes without complete DNA repair. J Biol Chem.

[33] Tang N, Wu Y, Ma J, Wang B, Yu R (2010). Coffee consumption and risk of lung cancer: a meta-analysis. Lung Cancer.

[34] Justinova Z, Ferré S, Barnes C, Wertheim CE, Pappas LA, Goldberg SR, Le Foll B (2009). Effects of chronic caffeine exposure on adenosinergic modulation of the discriminative-stimulus effects of nicotine, methamphetamine, and cocaine in rats. Psychopharmacology (Berl).

[35] Merighi S, Benini A, Mirandola P, Gessi S, Varani K, Simioni C, Leung E, Maclennan S, Baraldi PG, Borea PA (2007). Caffeine inhibits adenosine-induced accumulation of hypoxia-inducible factor-1alpha, vascular endothelial growth factor, and interleukin-8 expression in hypoxic human colon cancer cells. Mol Pharmacol.

[36] Boswell-Smith V, Spina D, Page CP (2006). Phosphodiesterase inhibitors. Br J Pharmacol.

[37] Nishijima H, Nishitani H, Saito N, Nishimoto T (2003). Caffeine mimics adenine and 2′-deoxyadenosine, both of which inhibit the guanine-nucleotide exchange activity of RCC1 and the kinase activity of ATR. Genes Cells.

[38] Valenzuela MT, Mateos S, Ruiz de Almodóvar JM, McMillan TJ (2000). Variation in sensitizing effect of caffeine in human tumour cell lines after gamma-irradiation. Radiother Oncol.

[39] Nehlig A, Daval JL, Debry G (1992). Caffeine and the central nervous system: mechanisms of action, biochemical, metabolic and psychostimulant effects. Brain Res Brain Res Rev.

[40] Kroemer G, Galluzzi L, Vandenabeele P, Abrams J, Alnemri ES, Baehrecke EH, Blagosklonny MV, El-Deiry WS, Golstein P, Green DR, Hengartner M, Knight RA, Kumar S, Nomenclature Committee on Cell Death 2009 (2009). Classification of cell death: recommendations of the Nomenclature Committee on Cell Death 2009. Cell Death Differ.

[41] Han A, Yang L, Frazier AB (2007). Quantification of the heterogeneity in breast cancer cell lines using whole-cell impedance spectroscopy. Clin Cancer Res.

